# Multiferroic Quantum Criticality in a Proper Ferroelectric Quasi‐one‐dimensional Ising Antiferromagnet

**DOI:** 10.1002/advs.77948

**Published:** 2026-09-29

**Authors:** Takayuki Nagai, Peisen Yu, Akitoshi Nakano, Tsuyoshi Kimura

**Affiliations:** ^1^ Quantum‐Phase Electronics Center (QPEC) and Department of Applied Physics University of Tokyo Bunkyo‐ku Tokyo Japan; ^2^ Department of Advanced Materials Science University of Tokyo Kashiwa Chiba Japan; ^3^ Department of Applied Physics Nagoya University Nagoya Aichi Japan

**Keywords:** ferroelectricity, multiferroic quantum criticality, multiferroic, phase transition, quantum critical points, quantum paraelectric

## Abstract

Quantum phase transitions are driven at absolute zero by quantum‐mechanical fluctuations, offering unique opportunities for the emergence of exotic phenomena ranging from unconventional superconductivity to quantum magnetism. In multiferroics, where magnetic and ferroelectric orders are cross‐correlated, this raises an intriguing possibility: a state of quantum criticality in which both fluctuations are intertwined. Here we show that the quasi‐one‐dimensional Ising antiferromagnet Sr_1−_ *_x_* Ba*_x_* Co_2_V_2_O_8_ hosts multiferroic quantum criticality through the coexistence of ferroelectric and magnetic quantum critical points (QCPs) tuned by independent non‐thermal parameters. Structural and dielectric analyses establish this system as a proper displacive‐type ferroelectric—an exceptionally rare feature among magnetic materials. Isovalent Ba substitution continuously suppresses the ferroelectric transition to zero temperature, producing a quantum paraelectric state with a signature *T* ^2^ scaling of the inverse permittivity. In this regime, a transverse magnetic field drives the system to a magnetic QCP. Their resulting coexistence gives rise to a pronounced magnetocapacitance, revealing a strong interplay between fluctuations in the lattice and spin systems. Our findings establish Sr_1−_ *_x_* Ba*_x_* Co_2_V_2_O_8_ as a unique platform for opening new avenues at the intersection of quantum criticality and multiferroicity.

## Graphical abstract

Sr_1−x_Ba_x_Co_2_V_2_O_8_ is established as a magnetic‐ion‐based proper displacive ferroelectric whose transition is continuously suppressed by Ba substitution to a ferroelectric quantum critical point. In the resulting quantum paraelectric regime, a transverse magnetic field induces magnetic quantum criticality, realizing multiferroic quantum criticality and revealing coupled spin and polar quantum fluctuations in a tunable multiferroic platform.

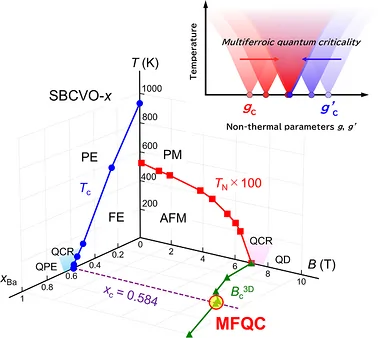

## 1 Introduction

Phase transitions are among the most universal phenomena in nature, ranging from the boiling and freezing of water to the emergence of superconductivity and structure formation in the early universe [1, 2]. In conventional thermal phase transitions, ordered phases are destabilized by thermal fluctuations. At absolute zero, where thermal fluctuations are absent, a classical description precludes phase transitions. Quantum mechanics changes this picture: quantum fluctuations associated with Heisenberg's uncertainty principle persist even at zero temperature and can drive phase transitions through a non‐thermal control parameter *g*, such as pressure, magnetic field, or chemical composition. When such a quantum phase transition is continuous, the boundary between distinct phases defines a quantum critical point (QCP). Although a QCP exists strictly at zero temperature, its influence extends over a broad finite‐temperature regime (Figure 1a), where the interplay between quantum and thermal fluctuations generates exotic physical properties that depart profoundly from conventional expectations [1]. Examples include the universal scaling of collective responses [3], non‐Fermi liquid behavior [4, 5], unconventional superconductivity [6, 7, 8] and emergent quantum symmetries [9].

**FIGURE 1:**
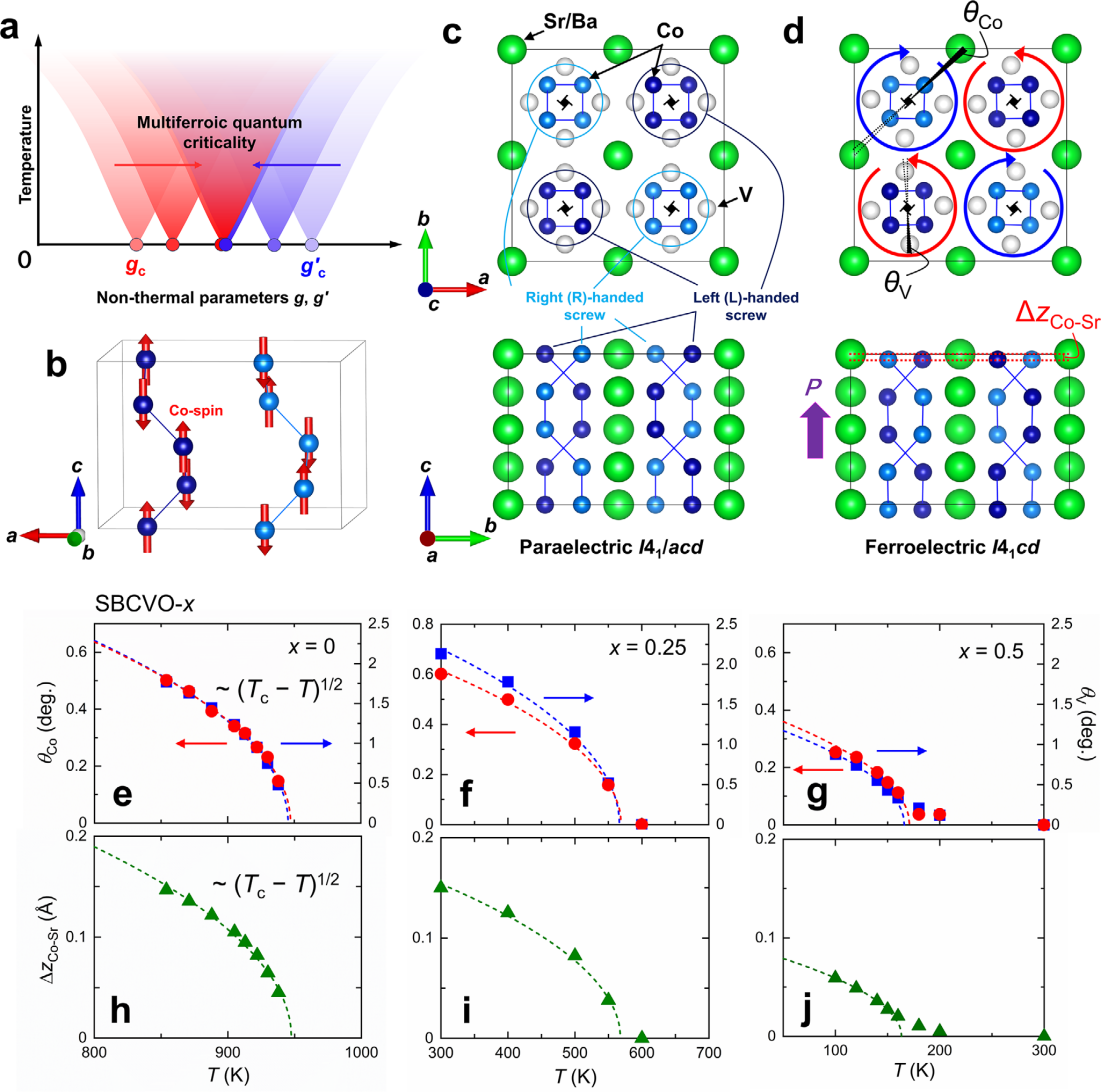
(a) Schematic of quantum‐critical regions emerging from magnetic and ferroelectric quantum critical points realized at non‐thermal control parameters *g* = *g*_c_ and *g* *'* = *g* *'*_c_, respectively. The coexistence of these two quantum critical points within a single system defines multiferroic quantum criticality. (b) Magnetic structure reported for SrCo_2_V_2_O_8_ [34]. Only the Co^2+^ screw chains are shown, and red arrows indicate the spin directions. For clarity, only two front chains are displayed; the two rear chains share the same spin arrangement as the front ones. (c,d) Crystal structures of SBCVO‐*x* in the (c) paraelectric and (d) ferroelectric phases. O ions are omitted in the upper panels, and V and O ions are omitted in the lower panels. *θ*_Co_ and *θ*_V_ denote the rotation angles of the Co and V positions about the screw axis. Δ*z*_Co‐Sr_ denotes the relative displacement between the Sr and Co sites along the *c* axis, representing the translational displacement of the screw chains. The ferroelectric structure of SBCVO‐*x* is induced by staggered rotational distortions coupled to the alternating arrangement of locally defined chirality. Temperature profiles of (e–g) *θ*_Co,V_ and (h–j) Δ*z*_Co‐Sr_ for (e,h) SBCVO‐0, (f,i) SBCVO‐0.25, and (g,j) SBCVO‐0.5. Dashed lines indicate fits to (*T*_C_ − *T*)^1/2^.

Quantum criticality has traditionally been described in terms of a single order parameter. Extending this framework to systems in which multiple quantum criticalities coexist offers a route to unique critical phenomena. A particularly intriguing case is multiferroic quantum criticality (MFQC)—where ferroelectric and magnetic QCPs coexist within a single system—which has been proposed as a platform for emergent phenomena through the coupling or competition between distinct quantum criticalities (Figure 1a) [10]. Ferroelectric quantum criticality is itself of broad interest, with signatures including non‐classical *T* ^2^ scaling of the inverse dielectric permittivity [11] and the potential enhancement of superconductivity via polar‐fluctuation‐mediated Cooper pairing [12, 13]. Phenomenological theory predicts that coupling to magnetic quantum criticality can modify the intrinsic scaling laws of both sectors. Despite this conceptual promise, experimentally accessible candidates for MFQC are practically limited to the (Eu,Ba,Sr)TiO_3_ system [14], apart from a related multiferroic critical end point observed in BiMn_2_O_5_, where a magnetic‐field‐induced metaelectric first‐order transition terminates near zero temperature [15]. This scarcity reflects not only the rarity of multiferroics [16] but also the difficulty of tuning multiple phase transitions to zero temperature without destroying long‐range order: upon tuning, transitions may vanish prematurely or give way to glassy behavior [17, 18, 19, 20, 21]. Identifying new materials in which multiple QCPs can be independently controlled is therefore essential for establishing MFQC as an accessible experimental platform.

Here, we propose the quasi‐one‐dimensional Ising quantum antiferromagnet Sr_1−_ *_x_* Ba*_x_* Co_2_V_2_O_8_ (SBCVO‐*x*) as a promising candidate material for hosting MFQC. The end members SrCo_2_V_2_O_8_ (SBCVO‐0) and BaCo_2_V_2_O_8_ (SBCVO‐1) are well‐established realizations of magnetic QCP. These tetragonal compounds contain screw chains of Co^2+^ running along the *c* axis (Figure 1c,d). Owing to the distorted octahedral crystal field and strong spin‐orbit coupling, the Co^2+^ moments are described by highly anisotropic effective *S* = 1/2 [22], exhibiting Ising antiferromagnetic (AFM) order below approximately 5 K with the easy axis along *c* (Figure 1b) [23, 24]. A magnetic field applied transverse to the screw chains maps the system onto the one‐dimensional transverse‐field Ising model (1DTFIM)—a prototypical model for quantum phase transitions and quantum criticality [25, 26, 27]. Indeed, transverse‐field‐induced magnetic QCPs, solitonic topological transitions and exotic dynamical excitation spectra described by the *E*_8_ Lie algebra have been demonstrated in this family [28, 29, 30, 31, 32]. Apart from its magnetism, polycrystalline SBCVO‐*x* was recently reported to exhibit ferroelectric order [33]. This ferroelectricity originates from a combination of local crystallographic chirality within each screw chain and an axial vector resulting from rotational distortions of the chains (Figure 1c,d). Moreover, the ferroelectric phase transition temperature (*T*_C_) depends strongly on composition *x*, suggesting that *T*_C_ is finely tunable through chemical substitution.

Motivated by this potential, we performed systematic structural, dielectric, and polarization measurements on single crystals of SBCVO‐*x*. We show that this system is a proper displacive ferroelectric despite containing magnetic ions, exhibiting highly anisotropic, divergently enhanced permittivity that obeys the Curie‐Weiss law. Near the critical composition *x*_c_, where the phase transition is tuned toward 0 K, the inverse permittivity follows the characteristic *T* ^2^ scaling, signaling a quantum paraelectric state. Crucially, a transverse magnetic field drives a magnetic QCP within this quantum paraelectric regime, realizing robust MFQC. We further observe a significant magnetocapacitance effect arising from the coupling between ferroelectric quantum criticality and spin correlations. These results establish SBCVO‐*x* as a pristine platform for investigating the interplay of multiple quantum criticalities, paving the way for exploring emergent phenomena unique to MFQC.

## 2 Results

### 2.1 Crystal Structure Analysis of SBCVO‐*x*

First, let us describe the room‐temperature crystal structures of the end members of the SBCVO‐*x* system. As shown in Figure 1c,d, SBCVO‐*x* consists of screw chains of CoO_6_ octahedra, forming an *antiferrochiral* structure, with neighboring chains of opposite chirality. In SBCVO‐0, the screw chains undergo clockwise or counterclockwise rotations whose periodicity follows the alternation of chirality (rounded arrows in the upper panel of Figure 1d). These rotations are coupled to a screw‐like translation relative to the Sr sublattice, resulting in the polar *I*4_1_ *cd* structure (lower panel of Figure 1d). This distortion is quantified by Co and V rotation angles (*θ*_Co_ and *θ*_V_) about the screw axes and by the relative displacement along the *c* axis between the Sr/Ba sublattice and the Co screw chains (Δ*z*_Co‐Sr_). In contrast, SBCVO‐1 lacks these distortions (i.e., *θ*_Co_ = *θ*_V_ = Δ*z*_Co‐Sr_ = 0), adopting the nonpolar *I*4_1_/*acd* structure (Figure 1c).

To investigate the structural evolution across the ferroelectric transition, we performed synchrotron X‐ray diffraction (XRD) measurements on single crystals of SBCVO‐*x* (*x* = 0, 0.25, and 0.5) grown by the laser floating‐zone technique (see Experimental Section). Figure 1e–j shows the temperature (*T*) profiles of *θ*_Co_, *θ*_V_, and Δ*z*_Co‐Sr_. For *x* = 0, although the measurements could only be performed up to 940 K due to experimental temperature limitations, all of these parameters decrease continuously with increasing *T*. Importantly, they exhibit a common temperature dependence that is well described by the characteristic (*T*_C_ − *T*)^1/2^ behavior expected for an order parameter of a continuous second‐order phase transition within mean‐field Landau theory. This behavior directly indicates that the coupled rotational and translational distortions of the screw chains, namely, the screw‐like mode, collectively constitute the order parameter. Because this screw‐like mode is symmetry‐equivalent to the spontaneous polarization [33], these results further support a proper ferroelectric transition in which the electric polarization itself serves as the primary order parameter. For *x* = 0.25 and 0.5, the corresponding structural parameters also follow the (*T*_C_ − *T*)^1/2^ dependence reasonably well, demonstrating that the second‐order nature of the ferroelectric transition is robust against Ba substitution. The transition temperatures obtained from the (*T*_C_ − *T*)^1/2^ fits to Δ*z*_Co‐Sr_ are ∼950, ∼568, and ∼163 K for *x* = 0, 0.25, and 0.5, respectively. These values agree well with the *T*_C_ values reported for polycrystalline samples [33], further supporting the assignment of these transitions as ferroelectric. For *x* = 0.5, a slight deviation from the (*T*_C_ − *T*)^1/2^ scaling is observed near the transition temperature. Because this composition does not exhibit relaxor behavior, as discussed below, this deviation may instead arise from a local distribution of transition temperatures caused by a slight spatial inhomogeneity in the Ba concentration *x* [35]. Details of the crystal structure analysis are provided in the Supporting Information (see Figure S6 and Tables S1 and S2). The absence of anomalies in differential thermal analysis (DTA) and volume discontinuities at *T*_C_ further supports the continuous nature of the phase transition (see Figures S1 and S2).

### 2.2 Dielectric and Ferroelectric Properties of SBCVO‐0.5

We next examine the dielectric and ferroelectric properties of SBCVO‐*x* single crystals. Because the electrical resistivity is insufficient for reliable dielectric and polarization measurements above room temperature, we focus on compositions for which Ba substitution suppresses *T*_C_ below room temperature. Figure 2a shows the *T* dependence of the relative dielectric permittivity *ε* *'* measured along the polar *c* axis for SBCVO‐0.5. Upon cooling, *ε* *'* increases divergently and reaches ∼1100 at *T*_C_ ≈ 130 K, consistent with the second‐order ferroelectric phase transition inferred from the crystal structure analysis. The dielectric loss (tan*δ*) remains below 0.1 even at the *ε* *'* maximum (see Figure S3a), indicating that the large dielectric response is intrinsic rather than dominated by extrinsic contributions, such as space charge redistribution [36, 37, 38] or thermally activated carriers [39, 40]. In contrast, *ε* *'* measured along the *a* axis exhibits only a kink‐like anomaly near *T*_C_, which is nearly two orders of magnitude smaller than the response along the *c* axis (see Figure S8). This significant anisotropy reflects the quasi‐one‐dimensional screw chain structure. Despite the 50% Ba substitution at the Sr site, the peak temperature of *ε* *'* is independent of measurement frequency, excluding relaxor‐like behavior induced by compositional randomness [41, 42] and demonstrating the robustness of ferroelectricity in SBCVO‐*x*.

**FIGURE 2:**
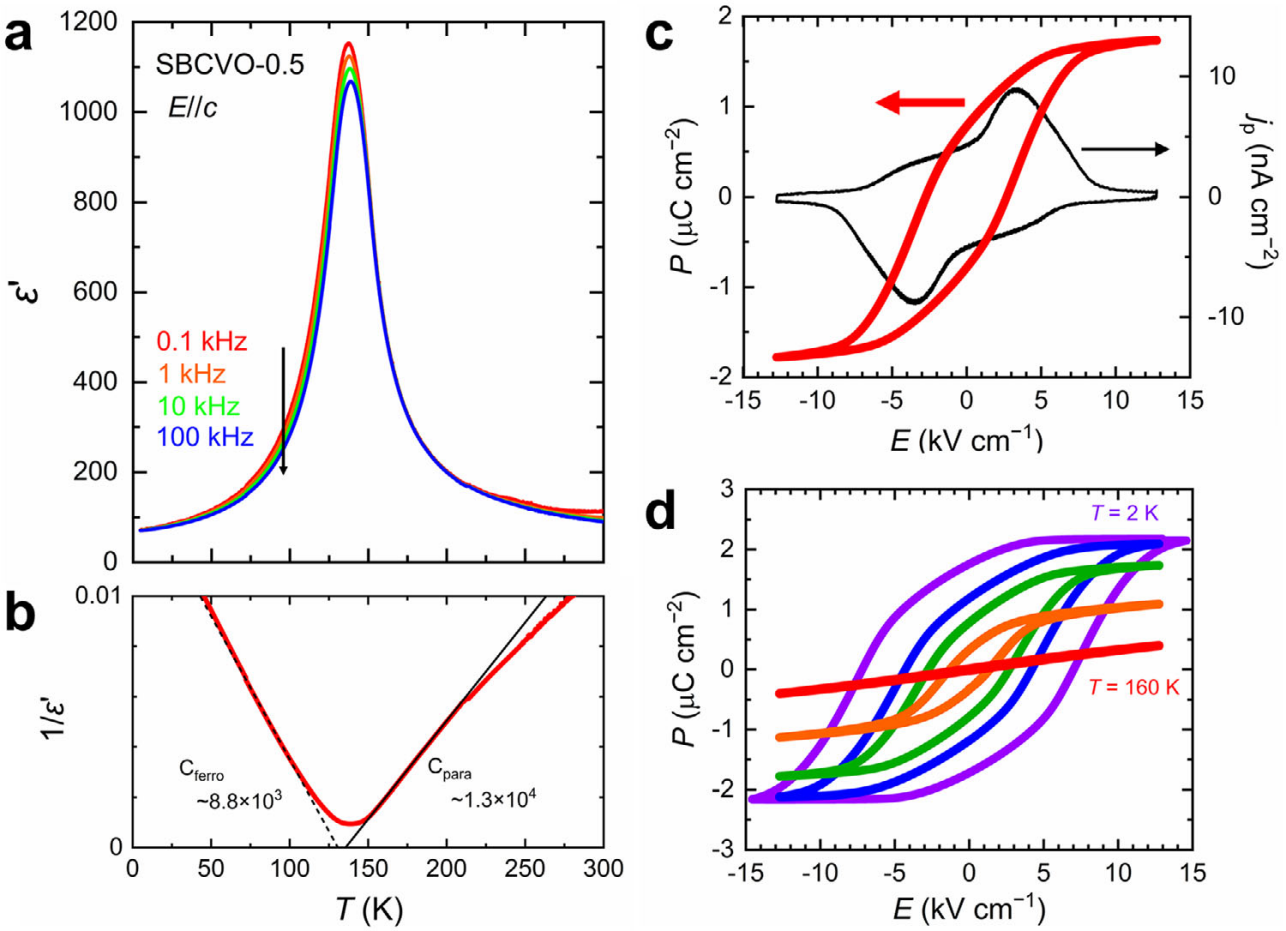
(a) Temperature (*T*) dependence of the permittivity *ε* *'* measured at 0.1, 1, 10 and 100 kHz. The electric field *E* was applied parallel to the polar *c* axis. The inset shows the *T* dependence of *ε* *'* measured with *E* applied along the *a* axis, perpendicular to the polar axis. (b) the inverse permittivity 1/*ε* *'* as a function of *T*. The solid and dashed lines indicate fits to the Curie–Weiss law, 1/*ε* *'* = |*T* – *T*_C_|/*C*, above and below the ferroelectric phase transition, respectively, where *C* is the Curie constant and *T*_C_ is the ferroelectric phase transition temperature. (c) Electric polarization (*P*) and polarization current density (*j*_p_) along the *c* axis as a function of *E* at 80 K for SBCVO‐0.5. The *j*_p_ measurements were performed under quasi‐DC conditions at frequencies below 0.1 Hz. (d) *P–E* hysteresis loops of SBCVO‐0.5 measured at *T* = 2, 40, 80, 120, and 160 K.

Figure 2b shows the inverse permittivity 1/*ε* *'* along the *c* axis for SBCVO‐0.5. In the vicinity of *T*_C_, 1/*ε* *'* varies linearly with *T*, following the Curie–Weiss law, 1/*ε* *'* ∝ |*T* – *T*_C_|. This establishes SBCVO‐0.5 as a canonical proper ferroelectric, in which electric polarization (*P*) itself serves as the order parameter. Such behavior is common in non‐magnetic proper ferroelectrics but exceedingly rare in ferroelectrics containing magnetic ions. For example, even in the prototypical magnetic ferroelectric BiFeO_3_, the high *T*_C_ and resulting poor insulation at elevated temperatures obscure a clear Curie–Weiss‐type divergence [43, 44], while chemical substitution readily suppresses ferroelectricity, making systematic control of *T*_C_ challenging [45, 46, 47]. Other representative magnetic ferroelectrics, including YMnO_3_ and Ca_3_Mn_2_O_7_, are improper ferroelectrics in which *P* is not the primary order parameter [48, 49, 50], and diluted magnetic ferroelectric systems typically show relaxor‐like behavior [51, 52]. SBCVO‐*x* therefore represents a rare magnetic‐ion‐based system showing clear Curie–Weiss behavior characteristic of an ideal proper ferroelectric. *T*_C_ is estimated to be 136 K from a Curie–Weiss fitting to the data above *T*_C_. Strictly speaking, Landau theory predicts a 1/*ε* *'* slope ratio of 2 between the ferroelectric and paraelectric phases for a second‐order proper ferroelectric phase transition [53, 54]. Deviations from this ideal ratio commonly arise from factors such as noncritical dielectric background [55, 56] and extrinsic domain‐wall dynamics [57]. Therefore, the observed deviation from the ideal 2:1 ratio in SBCVO‐0.5 does not contradict its proper ferroelectric nature.

To confirm switchable ferroelectricity, we measured the electric‐field (*E*) dependence of *P*. As shown in Figure 2c, SBCVO‐0.5 exhibits a well‐defined *P–E* hysteresis loop at 80 K (< *T*_C_). A distinct depolarization‐current peak clearly appears near the coercive field, confirming the intrinsic polarization switching rather than leakage‐related artighfacts [58]. No spontaneous polarization is detected at 160 K (> *T*_C_), whereas finite spontaneous polarization emerges below *T*_C_ (Figure 2d). The remanent polarization (*P*_r_) increases continuously on cooling and reaches ∼1.75 µC cm^−^^2^ at 2 K. The coercive field also increases on cooling but remains modest, ∼7 kV cm^−1^ at 2 K, substantially smaller than that of BiFeO_3_ (∼10^1^–10^2^ kV cm^−^^1^) [59, 60] and below that of widely used ferroelectrics such as SrBi_2_Ta_2_O_9_ (∼40 kV cm^−^^1^) [61]. These results indicate that *P* in SBCVO‐*x* can be readily switched by an external electric field.

### 2.3 Composition Dependence of Dielectric and Ferroelectric Properties

Figure 3a shows the *T* profiles of *ε'* for SBCVO‐*x* (0.45 ≤ *x* ≤ 0.6). With increasing *x*, the peak temperature of *ε* *'* shifts systematically to lower temperatures. Except for *x* = 0.45, the peak magnitude decreases with increasing *x*. The reduced response for *x* = 0.45 relative to *x* = 0.5 is most likely caused by degraded insulation near the transition temperature, which masks the intrinsic dielectric anomaly (see Figure S3b). Excluding this extrinsic contribution, the intrinsic dielectric response is expected to increase as *x* decreases. Figure 3b presents *ε* *'* at 4 K as a function of composition *x*. *ε* *'* increases with *x* and forms a sharp peak near *x* ≈ 0.6, suggesting a phase transition. Because this transition is controlled by chemical composition, a non‐thermal control parameter, it can be regarded as a quantum phase transition in the low‐temperature limit. As shown in the inset of Figure 3b, 1/*ε* *'* scales linearly with *x* around *x* ≈ 0.6 (1/*ε′ *∝ |*x* − *x*_c_|), in agreement with theoretical models incorporating quantum fluctuations near a QCP [62]. Linear extrapolation yields a critical composition of *x*_c_ ≈ 0.584. Consistently, the spontaneous polarization decreases toward zero as *x* approaches *x*_c_, as evidenced by the *P–E* loops and *P*_r_ (Figure 3c,d). Figure 3e summarizes the phase diagram obtained from single‐crystal XRD and dielectric measurements. SBCVO‐*x* thus provides a rare platform in which a high‐*T* second‐order ferroelectric phase transition is continuously suppressed by isovalent Ba substitution, enabling access to a ferroelectric QCP near *x*_c_ ≈ 0.584. For SBCVO‐0.59, whose Ba concentration *x* exceeds *x*_c_, no spontaneous polarization is observed even at 2 K, the lowest temperature accessible in our measurements, confirming the disappearance of ferroelectricity across the ferroelectric QCP (see Figure S7).

**FIGURE 3:**
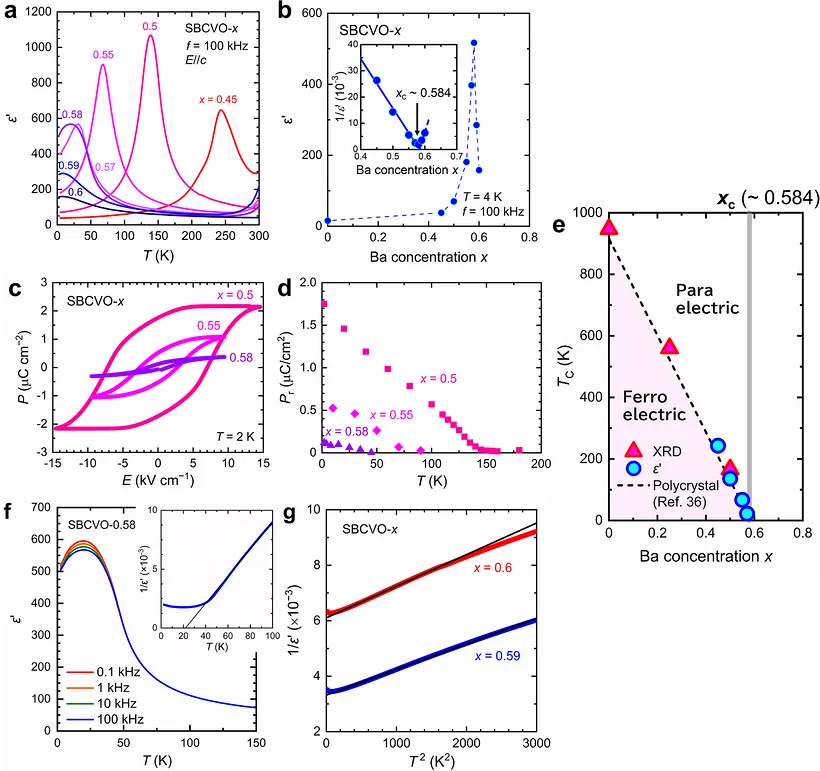
(a) Temperature (*T*) dependence of the permittivity *ε* *'* along the *c* axis for SBCVO‐*x* (*x* = 0.45, 0.50, 0.55, 0.57, 0.58, 0.59 and 0.60), measured at 100 kHz. (b) Composition dependence of *ε* *'* at 4 K, extracted from Figure 3a. The inset shows the inverse permittivity 1/*ε* *'* as a function of *x* at 4 K. Solid and dashed lines are fits to 1/*ε* *'* = *A*|*x* – *x*_c_| below and above the critical composition *x*_c_, respectively, where *A* is a proportionality constant. The fit yields *x*_c_ ≈ 0.584±0.005. (c) *P–E* hysteresis loops for SBCVO‐*x* (*x* = 0.5, 0.55, and 0.58) measured at 2 K. (d) The *T* dependence of the remanent polarization *P*_r_ extracted from the *P–E* hysteresis loops for SBCVO‐*x* (*x* = 0.50, 0.55, and 0.58). (e) Phase diagram of SBCVO‐*x*. Triangles and circles denote *T*_C_ determined from crystal structure analysis and dielectric measurements, respectively. The gray vertical line marks *x*_c_. The dashed line denotes the phase boundary reported in the polycrystalline samples [33]. (f) *T* dependence of *ε* *'* for SBCVO‐0.58 measured at 0.1, 1, 10 and 100 kHz. The inset shows the *T* dependence of 1/*ε* *'*. The black solid line denotes a Curie–Weiss fitting, 1/*ε* *'* = (*T* – *T*_C_)/*C*. (g) 1/*ε* *'* plotted against *T* ^2^ for SBCVO‐0.59 and SBCVO‐0.6 up to approximately 50 K. Black solid lines represent fits to the *T* ^2^ dependence.

### 2.4 Ferroelectric Quantum Criticality Near the Critical Composition

Given the second‐order nature of the ferroelectric phase transition, a ferroelectric QCP is expected near *x*_c_. Consistent with this picture, SBCVO‐0.58 (*x* < *x*_c_) exhibits a markedly broadened peak of *ε* *'* (Figure 3f). Although such broadening is often associated with relaxor behavior arising from substitutional disorder, the behavior of SBCVO‐0.58 is clearly distinct from that of relaxor ferroelectrics. In relaxors, the peak temperature of *ε* *'* typically shows a strong frequency dependence, and its *T* dependence deviates from the Curie–Weiss law [63, 64]. By contrast, SBCVO‐0.58 shows no detectable frequency dependence of the peak temperature up to at least 100 kHz, and 1/*ε* *'* above the peak follows the Curie–Weiss law (inset of Figure 3f). These features rule out relaxor behavior and indicate that the broadened anomaly reflects the suppression of long‐range ferroelectric order by quantum fluctuations. For compositions beyond *x*_c_ (SBCVO‐0.59 and SBCVO‐0.6), 1/*ε* *'* exhibits a non‐classical *T* ^2^ dependence (Figure 3g). This scaling is a hallmark of quantum paraelectrics near a ferroelectric QCP, as established in SrTiO_3_ and KTaO_3_ [11]. In general, the proximity to a QCP governs the strength of quantum fluctuations and the *T* range over which the *T* ^2^ regime persists [11]. The *T* ^2^ regime extends to higher temperatures in SBCVO‐0.59 than in SBCVO‐0.6, indicating that SBCVO‐0.59 lies closer to *x*_c_.

### 2.5 Magnetic Field‐Induced Magnetic QCP in SBCVO‐*x*

Although SBCVO‐0 and SBCVO‐1 are well‐established model systems for the 1DTFIM, the magnetic properties of their solid solutions have remained largely unexplored. Figure 4a shows the *T* dependence of magnetization (*M*) divided by a magnetic field (*B*) for SBCVO‐*x*, measured under *B* of 0.1 T applied along the *a* axis, perpendicular to the screw chains. An enlarged view of the region below 20 K is shown in Figure 4b. For SBCVO‐0, a sharp cusp associated with an AFM transition appears at *T*_N_ = 5.5 K. With Ba substitution, the overall *T* dependence remains essentially unchanged, indicating that the Ising AFM state is preserved across the solid‐solution series. Figure 4c shows the magnetic phase diagram derived from the *B* dependence of d*M*/d*B* (see Figure S4). In SBCVO‐0, a transverse magnetic field suppresses the three‐dimensional (3D) AFM order, giving rise to a 3D magnetic QCP. Near the critical magnetic field (*B*_c_^3D^), *T*_N_ follows *T*_N_ ∝ |*B*_c_^3D^ − *B*|^1/2^, yielding *B*_c_^3D^ ≈ 7.01 T, consistent with a previous study [27]. With increasing Ba content, *B*_c_^3D^ shifts systematically to higher fields while the topology of the phase diagram and the critical scaling remain essentially unchanged. Thus, the magnetic QCP is robust throughout SBCVO‐*x* and can be continuously tuned by chemical substitution. For *x* ≠ 0, *B*_c_^3D^ was tentatively estimated by fitting the data in the vicinity of the critical field to |*B*_c_^3D^ − *B*|^1/2^. As summarized in Figure 4d, *B*_c_^3D^ varies linearly with *x*, a trend that has also been noted in previous study [65]. Notably, near the ferroelectric critical composition *x*_c_ ≈ 0.584, applying the transverse critical field brings the magnetic quantum criticality into coexistence with ferroelectric quantum criticality, realizing an MFQC state in SBCVO‐*x*.

**FIGURE 4:**
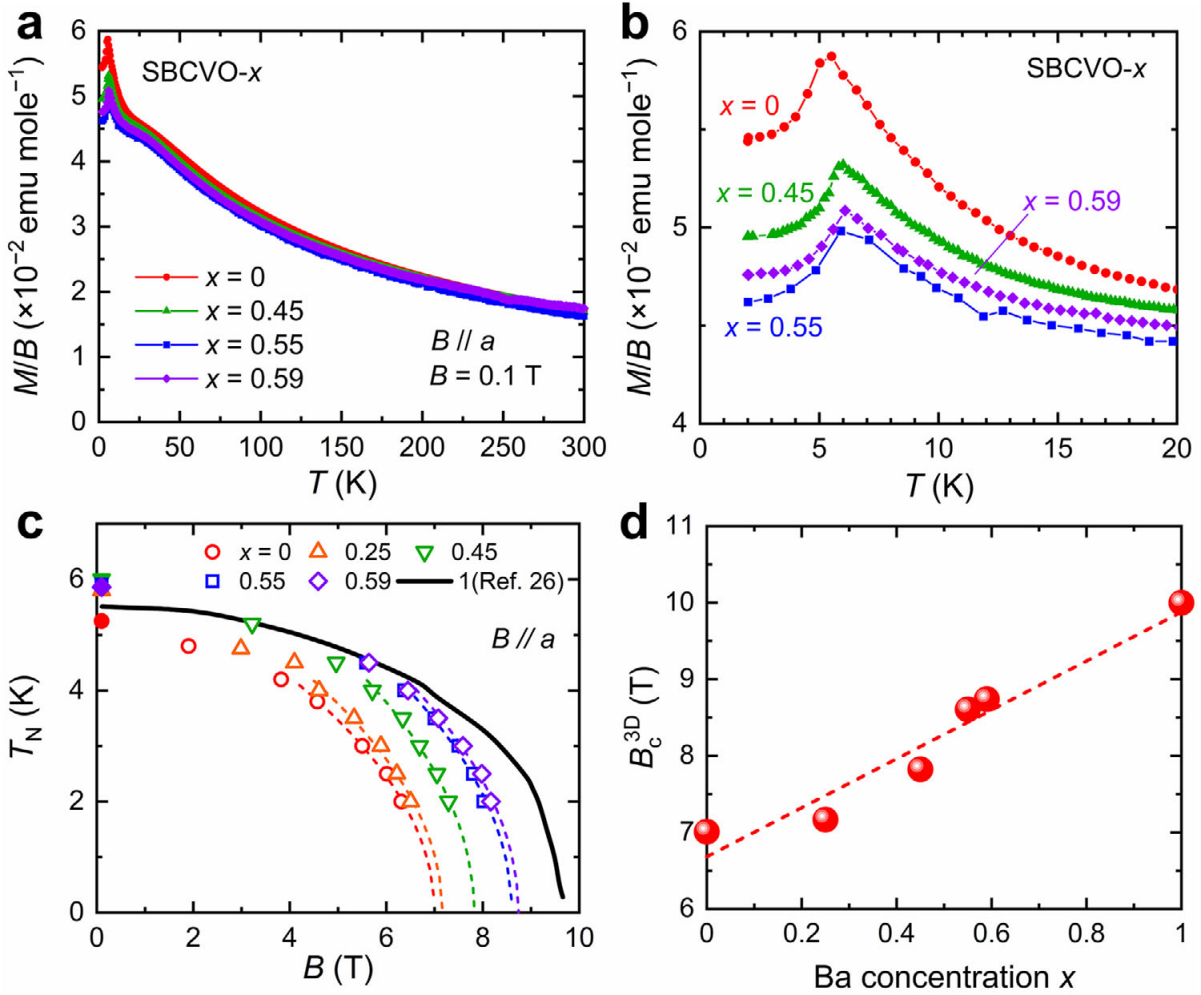
(a) Temperature (*T*) dependence of the magnetic susceptibility (*M*/*B*) for SBCVO‐*x* (*x* = 0, 0.45, 0.55, and 0.59) measured under a magnetic field (*B*) of 0.1 T applied along the *a* axis. (b) Enlarged view of the low‐temperature region below 20 K in panel Figure 4a. (c) Magnetic phase diagram of SBCVO‐*x* under a transverse magnetic field applied along the *a* axis. Filled symbols denote transition temperatures determined from *M–T* curves, whereas open symbols are obtained from field derivatives of *M–B* curves. Dashed lines represent fits to *T*_N_ = *β*(*B*_c_^3D^ − *B*)^1/2^. The black solid line shows the magnetic phase boundary of SBCVO‐1 compiled from a previous report [26]. (d) Ba concentration dependence of the critical field *B*_c_^3D^ obtained from the fits in Figure 4c. The red dashed line is a guide to the eye.

## 3 Discussion

We discuss the interplay between the ferroelectric and magnetic QCPs. In EuTiO_3_, where quantum paraelectricity coexists with magnetism, *ε* *'* scales with spin‐pair correlations through a non‐trivial coupling between optical phonons and spins [66]. Motivated by this analogy, we examine the dielectric and magnetic responses of the quantum paraelectric composition SBCVO‐0.59 under a transverse magnetic field. Figure 5a,b show the *T* dependences of *ε* *'* and *M/B*, respectively. At zero field, *ε* *'* exhibits a cusp‐like anomaly and a critical decrease at *T*_N_, indicating a coupling between optical phonons and spin‐correlations. This anomaly is substantially enhanced in the quantum paraelectric regime, where the *T*_C_ is suppressed close to *T*_N_, suggesting that the quantum fluctuation of polarization amplify the magnetodielectric coupling (see Figure S5). With increasing *B*, *T*_N_ is progressively suppressed and disappears at 9 T. Correspondingly, the dielectric anomaly shifts to a lower temperature and vanishes on the same field scale, closely tracking the magnetic response. In addition, *ε* *'* increases with *B*. As shown in Figure 5c, the positive magnetocapacitance (*ε* *'*(*B*)/*ε* *'*(0) − 1) grows approximately as ∼*B* ^2.7^ at 2 K and shows a pronounced change in slope near the transverse field where magnetization exhibits an anomaly, demonstrating that the dielectric response is tied to the evolution of magnetic correlations. These observations can be understood in terms of a magnetodielectric coupling with spin‐pair correlations, analogous to EuTiO_3_. Phenomenologically, *ε* *'* can be described as

$$\varepsilon \left(T,B\right)=\varepsilon \left(T,0\right)\left(1+\alpha \langle S_{i}\cdot S_{j}\rangle \right)$$

where 〈*S_i_* · *S_j_* 〉 is the spin‐pair correlation of Co spins. In the AFM state at zero field, 〈*S_i_* · *S_j_* 〉 is negative, whereas a transverse field drives spins toward a more field‐polarized configuration, rendering 〈*S_i_* · *S_j_* 〉 positive and thereby enhancing *ε* *'*. First‐principles calculations have suggested that ferroelectricity in the Sr*M*_2_V_2_O_8_ family is driven by a $\Gamma_{3}^{-}$ soft mode associated with screw motion [33]. The positive magnetocapacitance in SBCVO‐0.59 can therefore be attributed to the field‐induced softening of this polar mode through spin–phonon coupling. Although the underlying mechanism resembles that in EuTiO_3_, the quantitative response differs markedly. In EuTiO_3_, the Eu spins become fully polarized at ∼1.5 T, yielding a saturated permittivity change of ∼7% (Figure 6f) [66]. In contrast, SBCVO‐0.59 remains only partially polarized (∼0.4 µ_B_ per Co) even at 9 T, yet already exhibits a permittivity change of ∼5%. Although SBCVO‐*x* is a type‐I multiferroic with distinct microscopic origins for its ferroelectricity and magnetism, proximity to the ferroelectric QCP produces a strong magnetodielectric coupling. Similar spin‐correlation‐driven magnetocapacitance effects in several other systems, including *R*_2_Ti_2_O_7_ (*R* = rare earth) [67, 68] and (Se,Te)CuO_3_ [69] remain much lower than 1%. The exceptional responses of EuTiO_3_ and SBCVO‐0.59 therefore suggest that quantum paraelectricity near MFQCP amplifies magnetodielectric coupling, making pronounced magnetocapacitance a possible experimental signature of MFQC.

**FIGURE 5:**
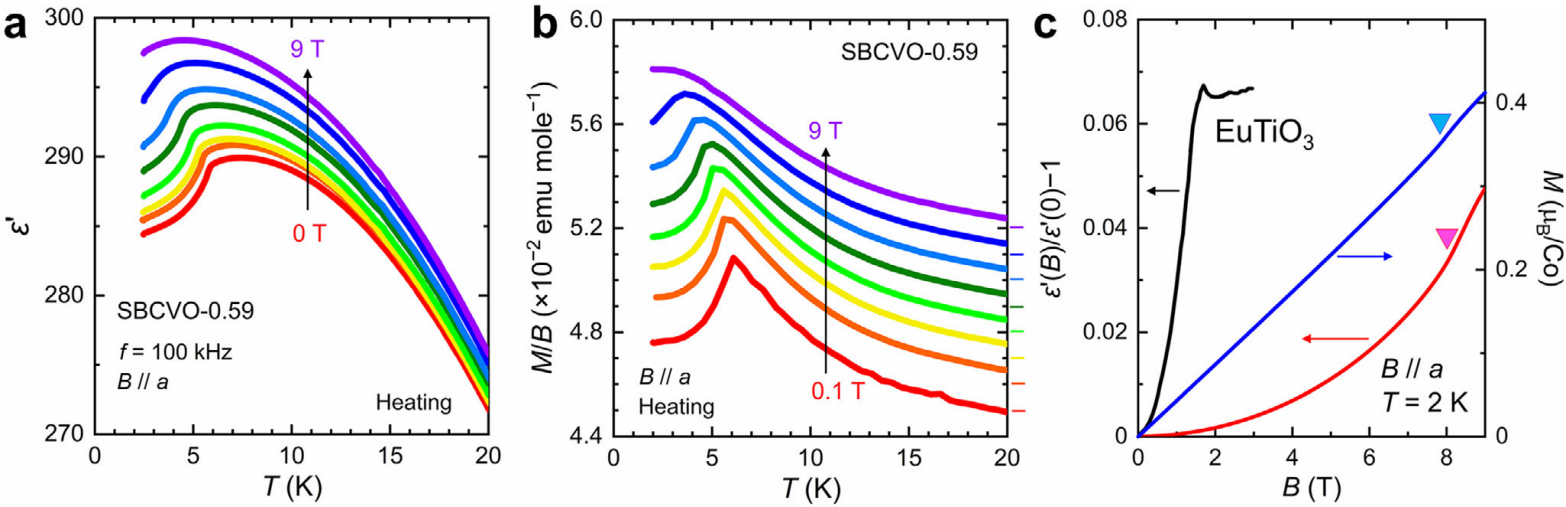
(a,b) Temperature (*T*) profiles of (a) *ε* *'* and (b) *M*/*B* for the quantum paraelectric composition SBCVO‐0.59 under transverse magnetic fields applied along the *a* axis (*B* = 0, 3, 4, 5, 6, 7, 8 and 9 T). The *M*/*B–T* curves in Figure 5b are vertically offset for clarity; the color scale indicates the offset relative to the 0 T curve. (c) *B* dependence of the relative change in permittivity, *ε* *'*(*B*)/*ε* *'*(0) − 1, that is, magnetocapacitance, and of *M* at 2 K under a transverse magnetic field. The black solid line shows the magnetocapacitance reported for EuTiO_3_ [66]. The inverted triangles indicate the magnetic transition point and the change in slope of *ε* *'*(*B*)/*ε* *'*(0) – 1.

**FIGURE 6:**
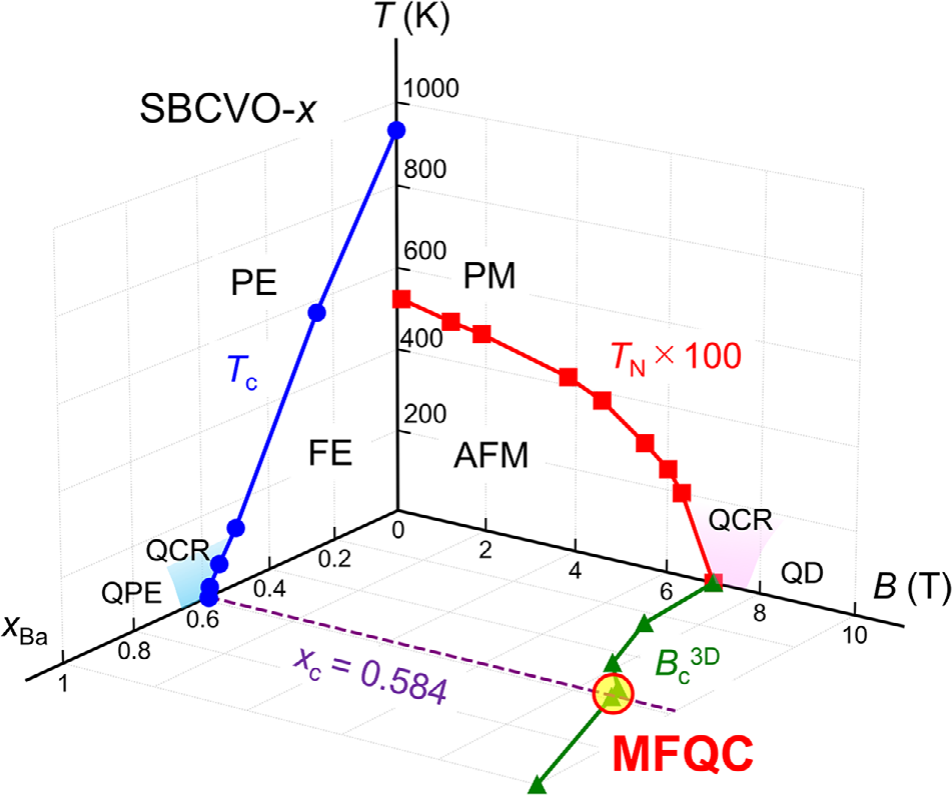
Temperature–composition–transverse magnetic field phase diagram of SBCVO‐*x*. The blue and red lines represent finite‐temperature phase boundaries of ferroelectric (FE) and antiferromagnetic (AFM) phases, respectively. The green line denotes the composition dependence of the 3D quantum critical field *B*_c_^3D^. Appropriate tuning of Ba concentration and transverse magnetic field brings the ferroelectric and magnetic quantum critical points into coexistence, thereby realizing multiferroic quantum criticality (MFQC).

Beyond the 3D magnetic QCP identified here, SBCVO‐0 and SBCVO‐1 also host a distinct 1D QCP associated with the 1DTFIM, accompanied by *E*8 symmetric excitations [31, 32], topological quantum phase transitions [29], and Bethe strings [70]. This 1D QCP has been experimentally established through NMR [27, 28, 31], AC susceptibility [27], and ultrasound measurements [28]. Exploring the regime where the magnetic 1D QCP intersects the ferroelectric QCP may reveal emergent phenomena arising from the interplay between ferroelectric quantum criticality and exotic magnetic excitations.

## 4 Conclusion

In the present study, we have demonstrated that the quasi‐one‐dimensional Ising antiferromagnet SBCVO‐*x* is a proper displacive ferroelectric in which electric polarization serves as the primary order parameter. Through the formation of a solid solution, the ferroelectric transition temperature can be tuned over an exceptionally wide range, enabling the realization of a ferroelectric QCP at the critical Ba concentration. In parallel, a transverse magnetic field drives the Ising AFM state toward a magnetic QCP, whose critical field is likewise tunable via composition. Therefore, this system provides a rare platform for MFQC in which ferroelectric and magnetic QCPs—independently controlled by composition and magnetic field, respectively—coexist within a single phase. In the quantum paraelectric regime, we observe a non‐trivial magnetodielectric response, indicative of a strong coupling between spins and polar phonons. Together with the exotic magnetic excitations in this family, our results establish SBCVO‐*x* as a quantum multiferroic platform for exploring coupled critical modes.

## 5 Experimental Section

### 5.1 Sample Preparation

Single crystals of Sr_1−_ *_x_* Ba*_x_* Co_2_V_2_O_8_ were grown by using a laser‐based floating‐zone (LFZ) furnace equipped with a five laser‐head design (Quantum Design LFZ1A). Polycrystalline rods were synthesized by a conventional solid‐state reaction. Stoichiometric amounts of SrCO_3_, BaCO_3_, CoO, and V_2_O_5_ were thoroughly ground. The mixture was heated in air at 850°C and 900°C for 24 h at each temperature, with intermediate grindings. The resulting powders were then pressed into rods and sintered at 900°C for 24 h. Single crystals were subsequently grown from these rods by the LFZ method at a typical growth rate of 0.6 mm h^−1^. The crystal growth was carried out in flowing air under ambient pressure. The obtained crystals were oriented using Laue X‐ray diffraction, and the plate‐shaped specimens were prepared.

### 5.2 Crystal Structure Analysis

The single‐crystal synchrotron X‐ray diffraction (XRD) experiments were conducted at beamline BL02B1 at SPring‐8 facility. The single crystals (100 × 50 × 20 µm^3^) were mounted on silica‐ or borosilicate‐glass capillaries using silver paste or epoxy adhesive. Diffraction data were collected with a Pilatus3 × 1 M CdTe area detector using a monochromatic X‐ray beam of wavelength 0.3430 Å. To perform temperature‐dependent measurements, the sample was cooled and heated in the range of 100–940 K using a N_2_ gas‐blowing device, with an estimated temperature uncertainty of ±10 K. Data reduction and lattice parameter refinement were carried out using the CrysAlisPro software suite. Structural parameters including atomic coordinates, anisotropic atomic displacements, and the batch scale factor were refined by using Jana2006 software.

### 5.3 Measurements of Dielectric and Magnetic Properties

The dielectric permittivity and electric polarization were measured using an LCR meter (E4980, Agilent) and an electrometer (6517B, Keithley), respectively, with a home‐built probe installed in a Physical Property Measurement System (PPMS, Quantum Design). The polarization measurements were performed under quasi‐DC conditions at frequencies below 0.1 Hz. The magnetic properties were measured using the AC Measurement System (ACMS) option of PPMS.

## Author Contributions

T.N. conceived the research project. T.N. and T.K. designed the experiments. T.N. and Y.P. prepared the single‐crystalline samples. T.N. and Y.P. performed the dielectric and magnetic measurements. A.N. performed the single‐crystal structural analysis. T.N. and Y.P. analyzed and interpreted the data. T.N. and T.K. wrote the manuscript with input from all authors.

## Conflicts of Interest

The authors declare no conflicts of interest.

## Supporting information




**Supporting File 1**: advs77948‐sup‐0001‐SuppMat.docx.

**Supporting File 2**: advs77948‐sup‐0002‐SuppMat.zip.

## Data Availability

The data that support the findings of this study are available from the corresponding author upon reasonable request.
