# Supplementary material for: Multiferroic Quantum Criticality in a Proper Ferroelectric Quasi‐one‐dimensional Ising Antiferromagnet
Source: Adv Sci (Weinh). 2026 Sep 29:e77948. Online ahead of print. doi: 10.1002/advs.77948 (PMC13624422; doi:10.1002/advs.77948)
Supplement: Supplementary file 1 [file ADVS-9999-e77948-s002.docx]

Supporting Information

**Multiferroic Quantum Criticality in a Proper Ferroelectric Quasi-one-dimensional Ising Antiferromagnet**

Takayuki Nagai,^*^ Peisen Yu, Akitoshi Nakano, Tsuyoshi Kimura

[*t-nagai@ap.t.u-tokyo.ac.jp](mailto:*t-nagai@ap.t.u-tokyo.ac.jp)

**Note S1 | Thermal properties of SBCVO-*x***

Figure S1 (a and b) show the differential thermal analysis (DTA) profiles for SBCVO-0 and SBCVO-0.25, respectively. No detectable mass change was observed over the measured temperature range for either sample (not shown). Furthermore, no discernible anomalies appear in the DTA curves near *T*_C_ (≈ 950 K for SBCVO-0 and ≈ 560 K for SBCVO-0.25). The absence of latent heat provides additional evidence that the ferroelectric phase transition in SBCVO-*x* is second order.

**Note S2 | Temperature evolution of the lattice parameters in SBCVO-0.25**

　Figure S2 (a and b) show the thermal evolution of the lattice parameters in SBCVO-0.25. Near 560 K, that is, *T*_C_ estimated from the data shown in Figs. 1f and 1i of the main text, the *c*-axis lattice parameter exhibits an anomaly, while the unit-cell volume remains continuous through the transition. This absence of a discontinuous volume change further supports the second-order nature of the ferroelectric phase transition in SBCVO-*x*.

**Note S3 | Low dielectric loss in SBCVO-0.5**

　Figure S3a shows the temperature dependence of the dielectric permittivity *ε*' and the dielectric loss (tan*δ*) measured along the polar *c* axis for SBCVO-0.5. Remarkably, despite the presence of magnetic Co^2+^ with partially filled *d* orbitals, SBCVO-0.5 exhibits an unusually low dielectric loss: tan*δ* remains below 0.1 even at the temperature where the *ε*' reaches its maximum. This indicates that the dielectric anomaly is intrinsic rather than dominated by leakage-related contributions.

**Note S4 | Reduced dielectric response in SBCVO-0.45 due to degraded insulation**

　Figure S3b displays the temperature dependence of *ε*' and tan*δ* measured for SBCVO-0.45. *ε*' exhibits a pronounced frequency dependence; at higher frequencies of 10 and 100 kHz, a peak-shaped dielectric anomaly appears near the expected ferroelectric phase transition temperature, whereas no clear peak is observed at lower frequencies of 0.1 and 1 kHz. In addition, tan*δ* shows a broad relaxation feature near the transition temperature, with a peak position that shifts markedly with frequency. Such dielectric relaxation is often associated with the thermal activation of leakage carriers. These observations suggest that dielectric relaxation overlaps with the ferroelectric phase transition in SBCVO-0.45, and that leakage-related degradation of the insulating properties masks the intrinsic dielectric response, resulting in a smaller apparent permittivity than expected from the composition dependence.

**Note S5 | Determination of magnetic phase diagram**

　A transverse magnetic field suppresses the antiferromagnetic Ising order in the SBCVO systems, inducing a phase transition that is visible in the magnetization response. Figure S4a shows the magnetization *M* for SBCVO-0 at 2 K as a function of magnetic field *B* applied along the *a* axis. A kink-like anomaly at ~6.3 T is clearly resolved as a peak in d*M*/d*B*, identifying the field-induced phase transition. Figure S4b displays d*M*/d*B* curves at various temperatures. The transition field shifts systematically with temperature; tracking these peak positions yields the magnetic phase diagram presented in Fig. 4c of the main manuscript.

**Note S6 | Magneto-dielectric coupling enhanced by coexistence of ferroelectric and magnetic fluctuation**

　Figure S5 (a and b) compare the dielectric response of SBCVO-0.5, a well-defined proper ferroelectric, with that of SBCVO-0.59, a quantum paraelectric. Both compositions exhibit a kink-like anomaly around the Ising antiferromagnetic transition temperature. However, the anomaly is more pronounced in SBCVO-0.59. This contrast indicates that the coupling between magnetic correlations and dielectric response is strongly enhanced in the quantum paraelectric regime. In SBCVO-0.5, *T*_C_ ≈ 130 K is far above the antiferromagnetic transition temperature *T*_N_ ~ 5.5 K, suggesting that ferroelectric fluctuations are largely quenched at *T*_N_. In SBCVO-0.59, however, the absence of long-range ferroelectric order is accompanied by quantum paraelectricity, implying that strong ferroelectric fluctuations survive around *T*_N_. The pronounced dielectric anomaly at *T*_N_ therefore suggests that magnetocapacitance in SBCVO-*x* is amplified by the coupling between quantum fluctuations of polarization and magnetic fluctuations.

**Note S7 | Reliability of single crystal structural analysis**

　 Figure S6 presents the experimental squared structure factors, $\left| F_{\mathrm{obs}} \right|^{2}$, obtained from the single-crystal structure analyses for SBCVO-0.25 and SBCVO-0.5, plotted against the corresponding calculated squared structure factors, $\left| F_{\mathrm{calc}} \right|^{2}$. Representative temperatures are shown for each composition: 600 K (paraelectric phase) and 300 K (ferroelectric phase) for SBCVO-0.25, and 300 K (paraelectric phase) and 100 K (ferroelectric phase) for SBCVO-0.5. For the refinement results at all measured temperatures, the data points lie close to the guide line ($\left| F_{\mathrm{obs}} \right|^{2}$ = $\left| F_{\mathrm{calc}} \right|^{2}$), demonstrating that the structural models reproduce the experimental data well and thereby confirming the high reliability of the single-crystal structure analyses. The X-ray diffraction data collection and structural refinement for SBCVO-0.25 and SBCVO-0.5 are summarized in Tables S1 and S2, respectively.

**Note S8 | Polarization measurement for quantum paraelectric SBCVO-0.59**

　 Figure S7 shows the *P*-*E* curves of SBCVO-0.58 and SBCVO-0.59, with a Ba concentration near the critical composition *x*_c_ ≈ 0.584, measured at 2 K, the lowest temperature accessible with our experimental setup. As shown in the main text, SBCVO-0.59 exhibits a *T*^2^ dependence of the inverse permittivity, characteristic of a quantum paraelectric state. Consistently, SBCVO-0.59 does not show spontaneous polarization in the *P*-*E* curve at 2 K. These results provide direct experimental evidence for the disappearance of ferroelectricity across the ferroelectric QCP.

**Note S9 | Strong dielectric anisotropy in SBCVO system**

Figure S8 shows the temperature dependence of the permittivity *ε*' along the *a* axis, perpendicular to the polar *c* axis, for SBCVO-0.5. While the *ε*' along the *c* axis exhibits a sharp, divergence-like peak near *T*_C_ ≈ 130 K, the *ε*' along the *a* axis shows only a kink-like anomaly around 130 K and is approximately two orders of magnitude smaller. This pronounced dielectric anisotropy is consistent with the strongly anisotropic crystal structure of the SBCVO system associated with its helical structural motif.

**Fig. S1. Thermal analysis at temperatures around the ferroelectric transition temperature *T*_C_ in BSCVO-0 and BSCVO-0.25.** (**a** and **b**) Differential thermal analysis (DTA) profiles for (a) SBCVO-0 and (b) SBCVO-0.25. The vertical bars denote *T*_C_ estimated from the data shown in Figs. 1h and 1i of the main manuscript.

**Fig. S2. Temperature variation of lattice parameters in SBCVO-0.25.** (**a** and **b**) Thermal evolution of (a) the lattice parameters *a* and *c* and (b) the unit-cell volume for SBCVO-0.25. The black solid line is a guide obtained by linear fits below the ferroelectric transition temperature. Grey vertical dashed line denotes the ferroelectric transition temperature estimated from the data shown in Figs. 1f and 1i of the main manuscript.

**Fig. S3. Dielectric permittivity and tan*δ* of SBCVO-0.5 and SBCVO-0.45.** Temperature dependences of (upper panel) dielectric permittivity *ε*' and (lower panel) dielectric loss tan*δ* measured along the polar *c* axis for (**a**) SBCVO-0.5 and (**b**) SBCVO-0.45 measured at 0.1, 1, 10, and 100 kHz.

**Fig. S4. Field-dependent magnetization of SBCVO-0 at various temperatures.** (**a**) Magnetization *M* as a function of magnetic field *B* applied along the *a* axis, perpendicular to the screw chains, at 2 K, including the corresponding field derivative d*M*/d*B*. (**b**) d*M*/d*B* curves measured at various temperatures.

**Fig. S5. Magneto-dielectric coupling of SBCVO-*x*.** Temperature profiles of dielectric permittivity *ε*' along the polar *c* axis of (**a**) SBCVO-0.5 and (**b**) SBCVO-0.59 measured at 100 kHz. The inverted triangles indicate the kink-like anomaly originating from the antiferromagnetic transition.

**Fig. S6. Results of single crystal structural refinement.** The observed squared structure factors, $\left| F_{\mathrm{obs}} \right|^{2}$, obtained from the single-crystal structural analyses for SBCVO-*x* (*x* = 0.25 and 0.5) are plotted against the corresponding calculated squared structure factors, $\left| F_{\mathrm{calc}} \right|^{2}$. Representative temperatures are shown for each composition: (**a**) 600 K (paraelectric phase) and (**b**) 300 K (ferroelectric phase) for *x* = 0.25, and (**c**) 300 K (paraelectric phase) and (**d**) 100 K (ferroelectric phase) for *x* = 0.5. The red solid lines represent guides corresponding to $\left| F_{\mathrm{obs}} \right|^{2}$ = $\left| F_{\mathrm{calc}} \right|^{2}$.

**Table S1. X-ray Data Collection and Refinement for SBMVO-0.25**

| Formula | Sr_0.75_Ba_0.25_Co_2_V_2_O_8_ (SBMVO-0.25) | |
| --- | --- | --- |
| Temperature | 300 K | 600 K |
| Wavelength (Å) | 0.32530 |  |
| Crystal system | Tetragonal |  |
| Space group | *I*4_1_*cd* | *I*4_1_/*acd* |
| Unit cell parameters | *a* = 12.3502(1) | *a* = 12.4112(2) |
|  | *c* = 8.4381(1) | *c* = 8.4597(2) |
| Volume (Å^3^) | *V* = 1287.04(3) | *V* = 1303.11(5) |
| *Z* | 8 | 8 |
| *F*(000) | 1652 | 1652 |
| Crystal size (µm^3^) | 90×60×20 µm^3^ |  |
| No. of unique reflectance | 12482 | 4411 |
| Data/restrains/parameters | 10827/ 0/ 61 | 2977/ 0/ 31 |
| Goodness of fit | 1.16 | 2.11 |
| Final *R*/*wR* factors | 0.0191/ 0.0237 | 0.0207/0.0382 |

**Table S2. X-ray Data Collection and Refinement for SBMVO-0.5**

| Formula | Sr_0.5_Ba_0.5_Co_2_V_2_O_8_ (SBMVO-0.5) | |
| --- | --- | --- |
| Temperature | 100 K | 300 K |
| Wavelength (Å) | 0.34300 |  |
| Crystal system | Tetragonal |  |
| Space group | *I*4_1_*cd* | *I*4_1_/*acd* |
| Unit cell parameters | *a* = 12.3378(1) | *a* = 12.3739(1) |
|  | *c* = 8.3983(1) | *c* = 8.4179(1) |
| Volume (Å^3^) | *V* = 1278.40(3) | *V* = 1288.89(3) |
| *Z* | 8 | 8 |
| *F*(000) | 1706 | 1698 |
| Crystal size (µm^3^) | 150×70×30 µm^3^ |  |
| No. of unique reflectance | 10560 | 5499 |
| Data/restrains/parameters | 9789/0/62 | 4672/0/32 |
| Goodness of fit | 1.14 | 1.84 |
| Final *R*/*wR* factors | 0.0182/0.0215 | 0.0214/0.0336 |

**Fig. S7. *P*-*E* curves near ferroelectric quantum critical point.** Electric polarization (*P*) and along the *c* axis as a function of electric field (*E*) at 2 K for SBCVO-0.58 and SBCVO-0.59. The *P*-*E* measurement was performed under quasi-DC conditions at frequencies below 0.1 Hz.

**Fig. S8. Temperature dependence of the permittivity along the *a* axis for SBCVO-0.5.** Temperature (*T*) dependence of the permittivity *ε*' measured at 10 kHz for SBCVO-0.5. The electric field (*E*) was applied parallel to the *a* axis perpendicular to the polar *c* axis.
